# Supplementary material for: Stress Relaxation Properties of Five Orthodontic Aligner Materials: A 14-Day In-Vitro Study
Source: Bioengineering (Basel). 2022 Jul 28;9(8):349. doi: 10.3390/bioengineering9080349 (PMC9405504; doi:10.3390/bioengineering9080349)
Supplement: Supplementary file 1 [file bioengineering-09-00349-s001.zip › bioengineering-1793107-supplementary.pdf]

File S1. Time–Temperature Superposition

Time–temperature superposition (TTS) is a principle of polymer physics. Ref. [26] The general idea behind this important concept is that viscoelastic phenomena, such as stress relaxation, occur faster when the material is kept at higher temperatures. Importantly, though, temperature will not modify the nature of the phenomenon: it acts only on the time by which it occurs. There are many practical applications of TTS. Ref. [20,27]

In this paper, we have considered the possibility of performing accelerated testing of stress relaxation. Stress relaxation is a viscoelastic phenomenon through which, when a material is kept under a constant state of deformation ε, the stress that is necessary to maintain such deformation σ decreases with time t (Figure S1). It is common to many materials: even metals relax their stresses, but they normally do so at high temperatures. With polymers, this behavior is evident also at room temperature.

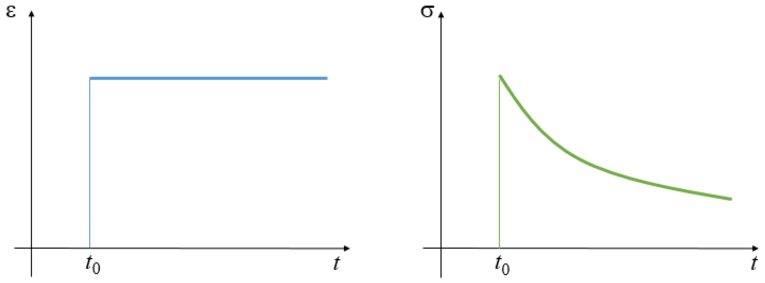

Figure S1. Description of stress–relaxation phenomenon.

Stress relaxation in polymers has a clear microstructural origin. From a molecular point of view, polymers are composed of long chains that are deeply entangled and loosely bond to one another and, moreover, are in perpetual movement due to temperature-induced oscillations. The application of a constant strain to such a material causes the entangled chains to move with respect to one another in such a way that weak intermolecular bonds are continuously broken and reformed in different positions. This is the phenomenon of relaxation at the molecular level and actually means that the material, despite being a solid, has a fluid-like behavior, and this is at the core of the word "viscoelastic", meaning that the behavior is hybrid, between that of a solid and that of a fluid.

TTS was first proposed in order to explain some of the viscoelastic properties of polymeric glasses. The key point of this principle is that the relaxation mechanisms occurring within polymers at the molecular level become faster as the polymer temperature is increased. As a consequence, it is not the amount of relaxed stress that changes, but the rate at which this relaxation takes place.

The principle is often conveniently used to perform accelerated viscoelastic testing of polymeric materials. Since a temperature increase determines an acceleration of molecular relaxation phenomena, the relaxation behavior at a certain temperature at a very large loading time can be approximated by the relaxation at a higher temperature but at a much smaller time. This idea is illustrated in Figure S2. In this figure, we can see a test in which the material is kept deformed under a constant displacement and the decreasing load or stress is continuously measured as a function of time. The solid curve illustrates the relaxation behavior of a certain polymer at temperature T_0 for a certain time t. This behavior is representative of what happens also at a lower temperature (say

T_1), but at longer times, as shown by the dashed curve. This can be represented by the following equation:

$$\sigma(t, T_0) = \sigma(\phi t, T_1) \tag{A1}$$

in which σ represent the relaxing stress and φ is the time shift factor. In fact, considering again Figure S2, notice that the decay in stress is the same in both curves, but the dashed curve (the one for the lower temperature) differs from the solid one by the scale factor φ that is applied to the time axis (i.e., the abscissa). In particular, the time scale becomes wider and moves slightly to the right.

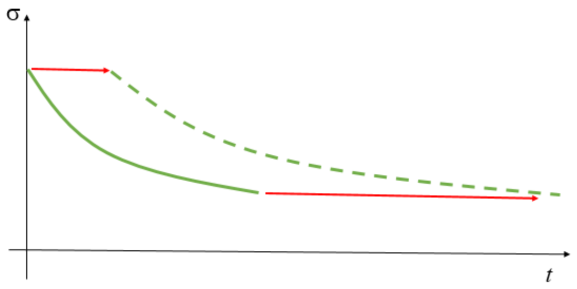

Figure S2. Shifting procedure according to TTS, where the dotted line describes the shifting of the curve after the test.

The usage of TTS as a means for performing accelerated viscoelastic testing is described and illustrated as follows for the Duran material (Figure S3). For better clarity, only the behavior up to 5 days is shown, but the procedure does allow one to reach 14–15 days, as pictured in the figures in the manuscript. The relaxation curve at 37 °C for 1 day is pictured in Figure S3 as the blue curve. Of course, the relaxation curve for the same material at 47 °C and 1 day is the red curve, relaxing much faster than the first one. By using the time–temperature superposition, the red curve must be multiplied by the scale factor φ; thus, it will move to the right (because $\varphi > 1$). In order to find the correct value for φ, care must be taken in order to ensure that the translated curve (blue dashed curve) and the curve at the lower temperature (the solid blue curve) have partial superposition, as pictured in Figure S3.

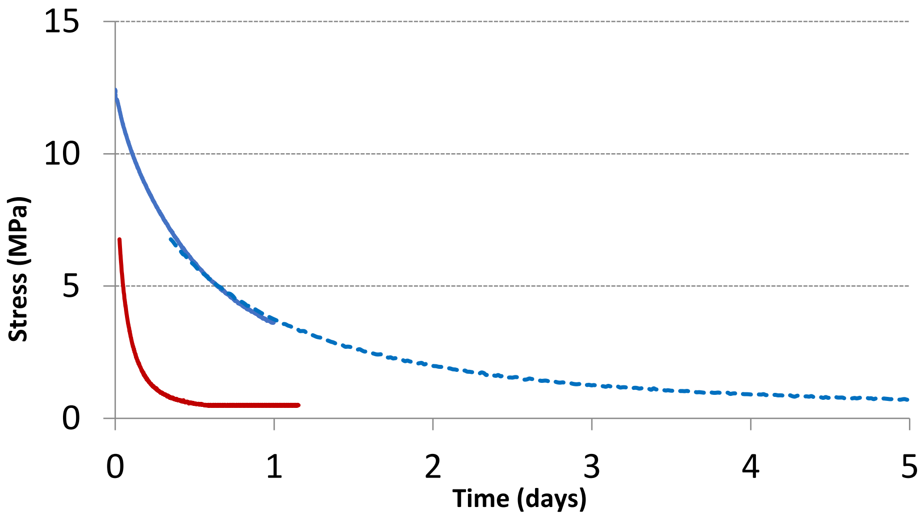

Figure S3. Shifting procedure applied to Duran aligner, where the red curve describes the tested material behavior at 37 °C; the blue line describes the tested material behavior at 47 °C, and the dotted line describes the shifting of the curve after the test at 47 °C.
